# Standard versus miniaturized percutaneous nephrolithotomy in pediatric patients: a systematic review and meta-analysis

**DOI:** 10.1590/1516-3180.2025.3486.29062026

**Published:** 2026-09-18

**Authors:** Fernanda Santos da Anunciação, João Lucas de Magalhães Leal Moreira, Caio Cezar Ferreira Fraga, Caio Vinicius Suartz, Ricardo Brianezi Tiraboschi, José Bessa, Roberto Iglesias Lopes

**Affiliations:** I Undergraduate Student, Escola de Medicina, Universidade Estadual de Feira de Santana (UEFS), Feira de Santana (BA), Brazil.; II Undergraduate Student, Escola de Medicina, Universidade Estadual de Feira de Santana (UEFS), Feira de Santana (BA), Brazil.; III Undergraduate Student, Escola de Medicina, Universidade Estadual de Feira de Santana (UEFS), Feira de Santana (BA), Brazil.; IV Assistant Professor, Urology Department, Northern Ontario School of Medicine, Thunder Bay (ON), Canada.; V Full Professor, Divisão de Urologia, Universidade Estadual de Feira de Santana (UEFS), Feira de Santana (BA), Brazil.; VI Full Professor, Divisão de Urologia, Universidade Estadual de Feira de Santana (UEFS), Feira de Santana (BA), Brazil.; VII Assistant Professor, Divisão de Urologia, Faculdade de Medicina, Universidade de São Paulo (FMUSP), São Paulo (SP), Brazil.

**Keywords:** Urology, Pediatrics, Urolithiasis, Nephrolithotomy, Percutaneous, Pediatric Endourology, Miniaturized Surgery, Percutaneous Renal Access, Perioperative Complications, Clavien-Dindo Classification, Renal Stone Surgery

## Abstract

**BACKGROUND:**

Although miniaturized percutaneous nephrolithotomy (mini-PCNL) is a less invasive alternative to standard percutaneous nephrolithotomy (PCNL), its comparative outcomes in pediatric patients remain unclear.

**OBJECTIVES:**

To compare the efficacy and safety of mini-PCNL versus standard PCNL in children with nephrolithiasis.

**DESIGN AND SETTING:**

Systematic review and meta-analysis conducted according to PRISMA guidelines, including randomized controlled trials (RCTs) and retrospective cohort studies of pediatric patients undergoing mini-PCNL or standard PCNL.

**METHODS:**

Major databases (Cochrane Library, PubMed/MEDLINE, and Embase) were searched through August 2024. The primary endpoint was the stone-free rate. Secondary endpoints included operative time, postoperative fever, and complications classified by the Clavien–Dindo system.

**RESULTS:**

Five studies (two RCTs and three retrospective cohort studies) involving 490 patients (46% underwent mini-PCNL) were included. Stone-free rates were comparable between the groups (relative risk [RR], 0.98; 95% confidence interval [CI], 0.929–1.041; p = 0.56; I^2^ = 0%). Mini-PCNL was associated with longer operative times (mean difference [MD], 7.5 min; 95% CI, 0.2–14.9; p = 0.04; I^2^ = 78%) but a lower risk of Clavien–Dindo grade I complications (RR 0.464; 95% CI 0.442–0.944; p < 0.05; I^2^ = 0%). No significant differences were observed in postoperative fever or Clavien–Dindo grade II–IV complications.

**CONCLUSION:**

Mini-PCNL and standard PCNL achieve comparable stone-free rates in pediatric patients. Mini-PCNL is associated with fewer minor complications, whereas standard PCNL is associated with shorter operative times. Both techniques demonstrate similar safety profiles with respect to major complications.

**CLINICAL TRIAL OR SYSTEMATIC REVIEW REGISTRATION:**

PROSPERO: CRD42024618399; available at:  https://www.crd.york.ac.uk/PROSPERO/view/CRD42024567890.

## INTRODUCTION

 Standard percutaneous nephrolithotripsy (PCNL) is the procedure of choice for treating complex and large kidney stones in both adults and children.^1^ However, bleeding requiring transfusion has been reported in up to 10% of cases and is associated with stone burden, operative time, the number of access tracts, and sheath size.^2-5^

 Recent innovations in standard PCNL have focused on reducing the size of access sheaths to minimize perioperative complications without compromising stone removal efficiency. Standard PCNL typically utilizes access sheaths ranging from 24F to 30F, whereas mini-PCNL employs smaller sheaths ranging from 14F to 20F, providing a less invasive alternative.^6,7^

 Meta-analyses comparing mini-PCNL (12–22F) with standard PCNL (> 22F) in adults have shown that both techniques achieve similar stone-free rates (SFRs). However, miniPCNL is associated with less blood loss, lower transfusion rates, and shorter hospital stays. No significant differences have been observed between the two approaches in overall complication rates.^6 -8^

 Evidence regarding the pediatric population remains limited, although recent studies have begun to address this gap. Therefore, this meta-analysis aimed to assess the efficacy and safety of mini-PCNL compared with standard PCNL for the management of kidney stones in children. 

## OBJECTIVE

 To compare the efficacy and safety of mini-PCNL versus standard PCNL in children with nephrolithiasis. 

## METHODS

### Literature search

 This systematic review and meta-analysis was conducted in accordance with the Cochrane Collaboration and the Preferred Reporting Items for Systematic Reviews and Meta-Analyses (PRISMA) statement guidelines. It was prospectively registered in the International Prospective Register of Systematic Reviews under registration number CRD42024618399. 

 The research question was developed using the Patient, Intervention, Comparator, Outcome, and Study framework: Which surgical approach, standard PCNL or mini-PCNL, yields a higher stone-free rate and better perioperative outcomes.^9^

### Eligibility criteria

 Randomized controlled trials (RCTs) and prospective or retrospective observational studies comparing standard PCNL and mini-PCNL in patients aged < 18 years with renal calculi were included if they reported at least one outcome of interest. Studies were excluded if they lacked a control group, included overlapping patient populations, enrolled patients without renal calculi, or evaluated a combination of other interventions or control groups. 

### Search strategy and data extraction

 PubMed, Embase, and the Cochrane Central Register of Controlled Trials were systematically searched through August 2024 using the following search strategy: (“children” OR “pediat ric” OR “adolescents” OR “patients under 18 years” OR “infants” OR “young patients” OR “youth” OR “neonates”) AND (“renal calculi” OR “kidney stones” OR “urolithiasis” OR “nephrolithi asis” OR “urinary calculi” OR “urinary stones”) AND (“mini PCNL” OR “mini percutaneous nephrolithotomy” OR “pediat ric mini percutaneous” OR “pediatric mini percutaneous neph rolithotomy” OR “minimally invasive PCNL” OR “mini neph rolithotomy” OR “micro PCNL” OR “super-mini percutaneous nephrolithotomy”) AND (“standard PCNL” OR “standard percutaneous nephrolithotomy” OR “conventional PCNL” OR “tradi tional PCNL” OR “regular PCNL”). 

 Two authors (C.F. and F.S.) independently extracted the data according to predefined eligibility criteria and performed the quality assessment. Disagreements were resolved by consensus. 

### Data extraction and endpoints

 All variables were entered into an Excel spreadsheet for analysis and cross-checked by another author (J.M. and F.S.). The mean and standard deviation (SD) were recorded for continuous variables whenever reported in the included studies. For variables reported as the median and interquartile range, the original data were converted to the mean and SD. 

 The recorded variables included the medium follow-up duration, number of patients, age, sex, study design, year of publication, stone size, stone-free rate, operative time, number of punctures, length of hospital stay, blood loss, transfusion rate, and complications stratified according to the Clavien–Dindo classification.^10^ Minor complications were defined as Clavien–Dindo grades I and II, whereas major complications were defined as Clavien–Dindo grades III–V. 

### Quality assessment

 The quality of the RCTs was assessed using the Cochrane Risk of Bias 2 tool for randomized trials, which evaluates studies across five domains: selection bias, performance bias, detection bias, attrition bias, and reporting bias. Each study was classified as having a low, high, or unclear risk of bias. For non-randomized studies, the Risk of Bias in Non-randomized Studies of Interventions tool was utilized. Two authors (C.F. and F.S.) independently assessed the risk of bias, and any disagreements were resolved by consensus. 

### Statistical analysis

 Treatment effects for continuous outcomes were estimated using the mean difference (MD) with 95% confidence intervals (CIs). Binary outcomes were analyzed using risk ratios (RR) with 95% CIs. For studies reporting continuous outcomes as medians and interquartile ranges, these values were converted to means and SD using established methods.^11^ Given the anticipated heterogeneity across studies, a random-effects model using the Mantel–Haenszel method was applied for all outcomes. Heterogeneity was assessed using the I^2^ statistic, with values of 0–25% indicating low heterogeneity, 25–50% indicating moderate heterogeneity, and > 50% indicating substantial heterogeneity.^12^ Statistical analyses were performed using R version 4.3.2 (R Foundation for Statistical Computing, Vienna, Austria). 

## RESULTS

### Study selection

**Figure 1:**
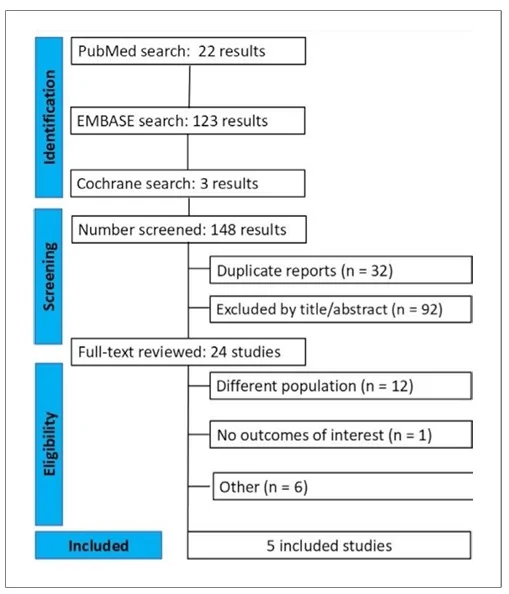
PRISMA flow diagram of the study selection process.

 A total of 148 records were identified through the electronic database search through September 30, 2024 (**Figure 1**), including 22 from PubMed, 123 from Embase, and three from the Cochrane Central Register of Controlled Trials. After removing 32 duplicates, 92 records were excluded based on their title and abstract because they were not relevant to the research question. Twelve articles were assessed for full-text eligibility. Of these, five were excluded because they did not involve the population of interest, one for not reporting the outcome of interest, and six for other reasons. Finally, two RCTs^13,14^ and three retrospective cohort studies comprising 490 patients^15-17^ were included in the systematic review and meta-analysis. 

### BASELINE CHARACTERISTICS OF THE INCLUDED STUDIES

**Table 1** summarizes the characteristics of the included studies. All studies were published in English between 2015 and 2024. The study populations were predominantly male, with participant ages ranging from 7 months to 11 years. 

**Table 1:** Baseline characteristics of the included studies

**Study**	**Year**	**Country**	**Study design**	**No of patients (PCNL/MPCNL)**	**Age, MPCNL/PCNL**	**Male, n (%) MPCNL/PCNL**	**Stone size, MPCNL/PCNL**
Akdogan	2024	Türkiye	Retrospective cohort	73/90	17.3 (7–24)/17.3 (8–24)^* #^	36 (49.3)/50 (55.6)	165.4 (50–600)/151.6 (50–800) mm^2^
Karkin	2024	Türkiye	Retrospective cohort	32/41	9.3 (4.1)/10.1 (5.4)^&^	19 (59.3)/29 (70.7)	2.1 (1.2)/2.3 (1.4) mm ^2^
Kumar	2023	India	RCT	25/25	9.4 (2.6)/10.4 (2.26)^&^	18 (72)/16 (64)	18.6 (2.56)/20.2 (3.58) mm^2^
Mahmood	2019	Iraq	Retrospective cohort	59/75	6.91 (4.982)/6.20 (4.138)^&^	31 (52.5)/51 (68)	1.9 (1.162)/2.2 (1.424) cm
Song	2015	China	RCT	35/35	20.3 (6.35)/20.10 (7.22)^#^	–	2.32 (1.07)/2.4 (1.39) cm

* Age reported as median (minimum–maximum).

# Age reported in months.

& Age reported in years.

MPCNL, miniaturized percutaneous nephrolithotomy; PCNL, percutaneous nephrolithotomy; RCT, randomized controlled trial.

### Pooled analysis of all studies: perioperative outcomes—operative time

**Figure 2:**
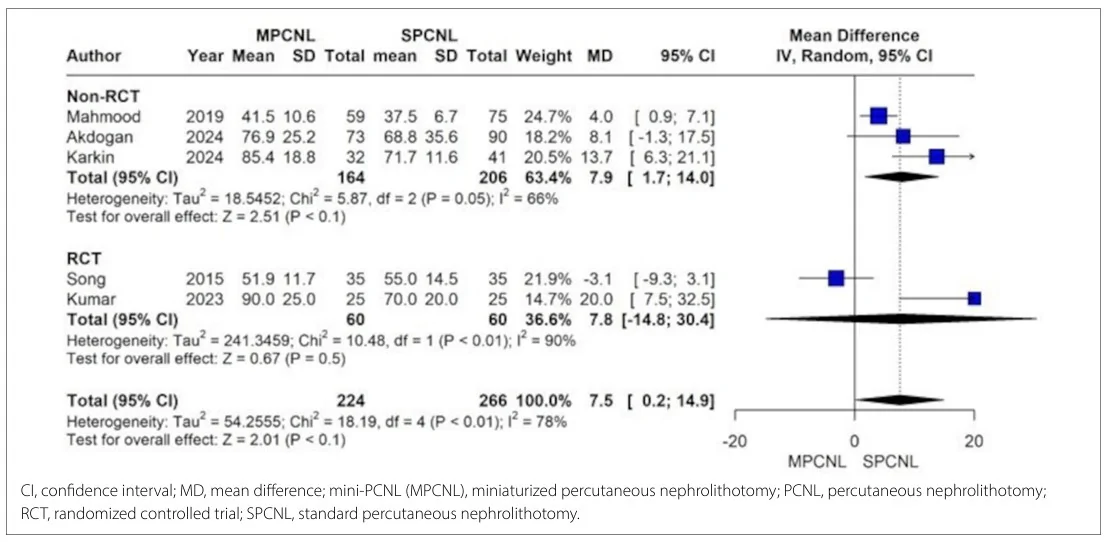
Forest plot of operative time comparing mini-PCNL and standard PCNL.

**Table 2** summarizes the perioperative outcomes comparing mini-PCNL and standard PCNL. Operative time was reported in all included studies. Kumar et al.^14^ reported the longest operative times in both the mini-PCNL and standard PCNL groups. The mean operative time was 90 min in the mini-PCNL group and 70 min in the standard PCNL group. This 20-min difference was statistically significant (p < 0.05), indicating a longer operative time with mini-PCNL. Conversely, Mahmood et al.^17^ reported the shortest operative times. The mean operative time was 41.53 min in the mini-PCNL group and 37.52 min in the standard PCNL group. This approximately 4-min difference was not statistically significant (p = 0.073), suggesting comparable operative times between the two techniques. The pooled analysis demonstrated that mini-PCNL was associated with a significantly longer operative time compared with standard PCNL (MD, 7.5 min; 95% CI, 0.2–14.9; p = 0.04; I ^2^ = 78%). However, subgroup analysis showed that this difference was mainly driven by nonrandomized studies, whereas randomized controlled trials did not demonstrate a statistically significant difference. Sensitivity analysis did not identify any source of heterogeneity (**Figure 2**). 

**Table 2:** Perioperative outcomes of mini-PCNL vs. standard PCNL

**Study (Year)**	**Group**	**SFR (%)**	**Operative time (min)**	**Hemoglobin/Hematocrit decrease (g/dL)**	**Transfusion rate (%)**	**Minor complications (Clavien–Dindo grades I–II)**	**Major complications (Clavien–Dindo grades ≥III)**
2024	MPCNL	89	74.8^*^	−1.7% (Hct)^*^	1.4	19.2%	4.1%
	PCNL	90.8	62.8	−1.7% (Hct)	3.3	17.7%	5.5%
2024	MPCNL	81.2	85.4^*^	0.9^*^	3.1	21.9%	0
	PCNL	85.4	71.7	1.6	4.9	29.3%	0
2023	MPCNL	88	90^*^	1.1^*^	4	44%	0
	PCNL	92	70	1.7	8	72%	4%
2019	MPCNL	89.5	41.53	0.35	1.7	18%	0
	PCNL	94.7	37.52	0.57^*^	5.3	44%	0
2015	MPCNL	97.8	51.97	0.9	0	0	0
	PCNL	94.3	55	0.8	0	0	0

* Indicates a statistically significant difference (p < 0.05) between the mini-PCNL and standard PCNL groups for the corresponding outcome in the original study.

Hct, hematocrit; MPCNL, miniaturized percutaneous nephrolithotomy; PCNL, percutaneous nephrolithotomy; SFR, stone-free rate.

### Complications according to the Clavien–Dindo classification

 The Clavien–Dindo classification of complications was assessed in four studies. Unlike the other studies, which used the ClavienDindo classification, Song et al.^13^ did not report complications using this system. Minor complications (Clavien–Dindo grade I–II) occurred more frequently in the standard PCNL groups, with incidences ranging from 17.7%–72%, compared with 11%–44% in the mini-PCNL groups. Among these complications, fever was the most common, affecting 15.5%–19.5% of patients undergoing standard PCNL compared with 5.7%–15.6% of those undergoing mini-PCNL. Hematuria occurred in 1.7%5.3% of patients in the standard PCNL groups and 1.4%–3.1% of those in the mini-PCNL groups. This difference reached statistical significance for grade I complications (p = 0.047), supporting a more favorable safety profile for the mini-PCNL approach. Regarding major complications (Clavien–Dindo grade ≥ III), standard PCNL was associated with a higher frequency of serious adverse events, with rates ranging from 1.1%–5.5%, whereas mini-PCNL showed incidences ranging from 0%–4.1%. Among the most severe complications, ureteral avulsion occurred in 1.1% of patients undergoing standard PCNL, and nephropleural fistula was reported in 4% of procedures. Although rare, these complications occurred exclusively or predominantly in the standard PCNL groups, suggesting that mini-PCNL may reduce the risk of serious surgical complications. 

**Figure 3:**
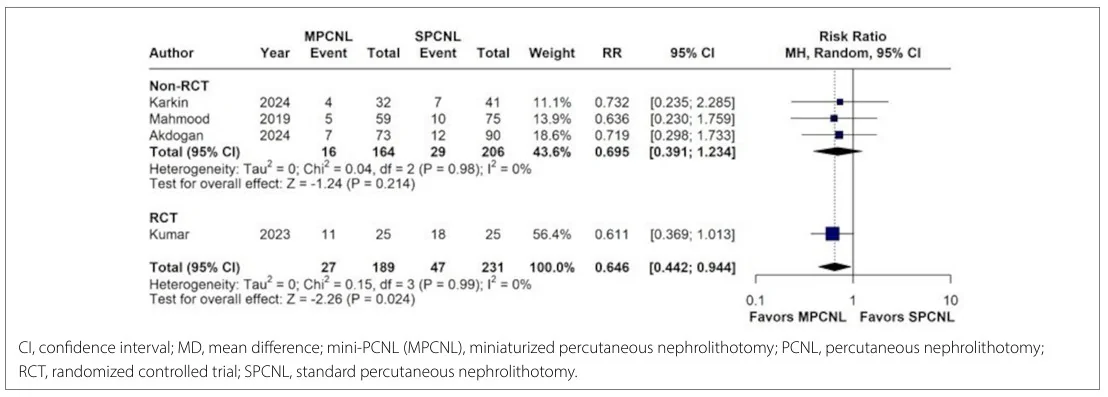
Forest plot of Clavien–Dindo grade I complications comparing mini-PCNL and standard PCNL.

 The pooled analysis demonstrated a significant difference between the groups for grade I complications (**Figure 3**: RR, 0.464; 95% CI, 0.442–0.944; p < 0.05; I^2^ = 0%). However, no significant differences were observed between the groups for grades II, III, and IV (p > 0.05 for all comparisons). 

### Postoperative fever

**Figure 4:**
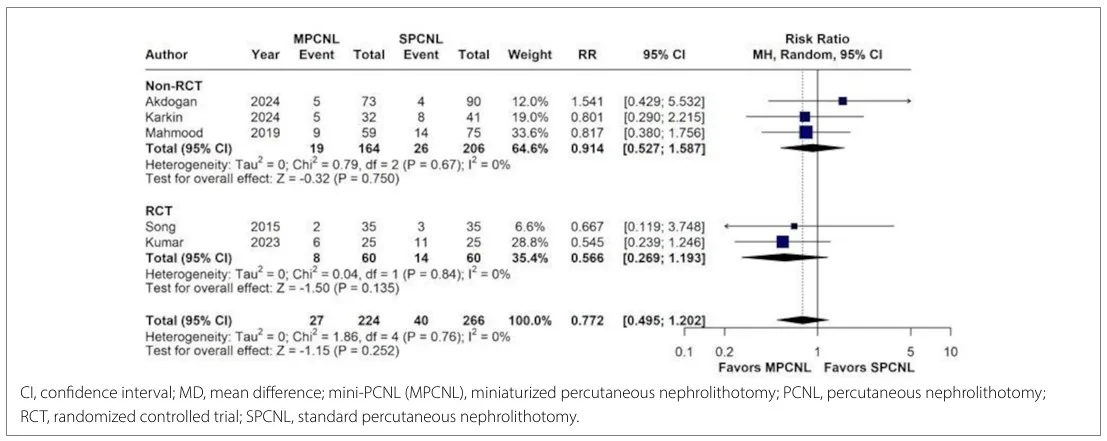
Forest plot of postoperative fever comparing mini-PCNL and standard PCNL.

 Postoperative fever was assessed in four studies. These studies included a total of 224 patients in the mini-PCNL group and 266 in the standard PCNL group. The pooled analysis showed no significant differences between the groups (**Figure 4**: RR, 0.772; 95% CI, 0.495– 1.202; p = 0.252; I^2^ = 0%). 

### Stone-free rate

 SFR was assessed in all included studies, although its definition varied. Most studies defined SFR as the absence of residual stones or the presence of clinically insignificant fragments. Mahmood et al.^17^ evaluated treatment success without specifying the allowable residual fragment size, focusing instead on complete stone removal. Kumar et al.^14^ and Karkin et al.^16^ defined SFR as residual fragments measuring <3 mm, whereas Akdogan et al.^15^ used a stricter cutoff of <2 mm. Song et al.^13^ considered residual fragments of up to 4 mm as stone-free. These varying definitions reflect differences in the assessment of SFR across studies. 

**Figure 5:**
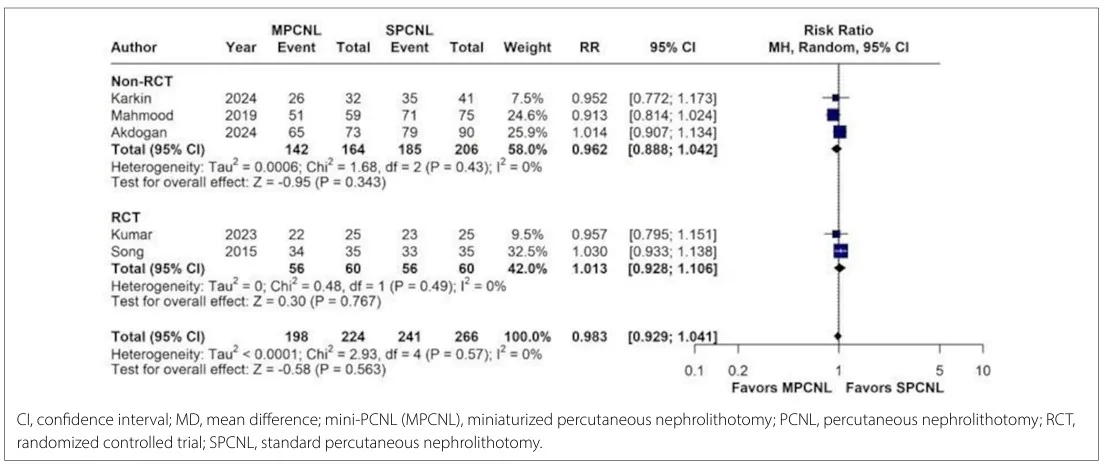
Forest plot of stone-free rate comparing mini-PCNL and standard PCNL.

 Song et al.13 reported the highest SFR, with the rates of 97.14% in the mini-PCNL group and 94.29% in the standard PCNL group. Karkin et al.16 reported the lowest SFR, with rates of 81.25% and 85.37%, respectively. The pooled analysis showed no significant difference between the groups (**Figure 5**: RR, 0.98; 95% CI, 0.929-1.041; p = 0.56; I^2^ = 0%). 

### Quality assessment

 The quality assessment indicated a low risk of bias across all assessed domains (**Supplementary Figures 1 and 2**). 

 The analysis of the studies included in this meta-analysis identified several methodological limitations that warrant consideration, although they do not undermine the overall validity of the findings. The main limitation was incomplete reporting in some studies, particularly regarding follow-up losses (D3) and outcome selection criteria (D5), resulting in an unclear risk of bias classifications for these domains. 

 The retrospective observational studies exhibited methodological characteristics requiring consideration, including challenges in controlling for confounding variables and potential selection bias, particularly in the study of Mahmood et al.^17^ The study by Karkin et al.^16^ was notable for its 20% loss to follow-up. Variability was also observed among the studies in outcome measurement methods and the reporting of statistical procedures. 

## DISCUSSION

 The main findings of this study can be grouped into three key areas: efficacy, safety, and operative aspects. 

 The most relevant finding was the absence of a significant difference in SFR between the two techniques analyzed (RR, 0.98; 95% CI, 0.929–1.041; p = 0.56). This finding is consistent with that of Qin et al.,^18^ who also reported no significant difference in SFR between miniPCNL and standard PCNL (RR, 1.01; 95% CI, 0.98–1.04; p = 0.57). 

 Mini-PCNL demonstrated a more favorable safety profile, with a lower incidence of Clavien–Dindo grade I complications,^10^ such as postoperative pain and transient fever (RR, 0.464; 95% CI, 0.4420.944; p < 0.05), possibly because of the reduced trauma associated with the use of smaller-caliber instruments. In contrast, no significant difference was observed in major complications (grades II–IV), including significant bleeding or the need for reoperation. Standard PCNL was associated with a significantly shorter operative time (MD, 7.5 min; p = 0.04), which may be related to greater familiarity with conventional instruments. However, this advantage was accompanied by a greater need for multiple punctures (RR, 2.946; 95% CI, 1.175–7.389; p < 0.05), a factor that may increase the risk of bleeding and renal parenchymal injury 

 Although mini-PCNL required a slightly longer operative time, it was less invasive, requiring fewer additional access tracts. 

 The analysis of postoperative fever revealed no significant difference between the two techniques (RR, 0.772; 95% CI, 0.495–1.202; p = 0.252; I^2^ = 0%), a finding that is consistent with that reported by Qin et al.,^18^ who also found no significant difference (RR, 1.22; 95% CI, 0.97–1.51; p = 0.08). 

 Additionally, no clinically relevant differences were observed in secondary outcomes, including hemoglobin loss, length of hospital stay, and the need for double-J catheter usage. Thus, although miniPCNL was associated with a lower rate of minor complications, both techniques demonstrated comparable outcomes with respect to postoperative fever, hemorrhagic outcomes, and immediate postoperative recovery. Therefore, the choice between the two approaches should consider the patient’s clinical profile, the desired operative time, and the goal of minimizing less clinically significant interventions. 

 Despite the promising results, some limitations should be considered. 

 The inclusion of studies with different designs, two RCTs^13,14^ and three retrospective cohorts,^15-17^ limits the direct comparability of the data. While the RCTs^13,14^ provide better control of potential bias, retrospective studies^15-17^ relied on medical records, which may be incomplete or inconsistent. Specifically, Mahmood et al.^17^ did not clearly describe the participant selection criteria, and Karkin et al.^16^ reported a high loss to follow-up rate (20%), which may have compromised the internal validity of the findings. 

 Five studies, including a total of 490 patients, were included, with an unequal distribution between the mini-PCNL (46%) and standard PCNL groups. The limited number of RCTs reduces the statistical power and generalizability of the findings. Several methodological limitations hindered the standardization of the results, including the lack of clearly defined exclusion criteria, unclear outcome assessment protocols, and inconsistent application of ClavienDindo classification (not reported in Song et al.^13^). Technical differences, such as instrument diameter (14F–20F in mini-PCNL vs. 24F–30F in standard PCNL) and the use of fluoroscopic versus ultrasonographic guidance, were also not controlled and may have influenced outcomes such as bleeding and operative time. 

 Important clinical variables, such as patient age and stone burden, were not explored despite their potential influence on the outcomes. Furthermore, most studies were conducted in countries with distinct epidemiological characteristics and surgical practices (Türkiye, India, Iraq, and China), which may limit the generalizability of the findings to other populations. 

 Despite these limitations, mini-PCNL and standard PCNL demonstrated comparable efficacy for the treatment of pediatric kidney stones. 

 An important observation is that none of the included studies were conducted in high-income countries. This may reflect regional differences in case volume, the adoption of mini-PCNL techniques, the availability of specialized pediatric stone centers, and publication patterns. Countries with a higher burden of pediatric urolithiasis and greater experience with PCNL may therefore be disproportionately represented in the available literature. Consequently, although the pooled results suggest comparable efficacy and safety between mini-PCNL and standard PCNL, their generalizability to high-income healthcare settings remains uncertain and warrants further investigation. 

 Mini-PCNL was associated with a 53% reduction in mild complications, whereas standard PCNL was associated with a significantly shorter operative time, albeit with a greater need for multiple punctures. In contrast, no significant differences were observed in major complications, postoperative fever, or other secondary outcomes. Therefore, the choice of technique should be individualized, taking into account the patient’s clinical characteristics, stone characteristics, the institutional context, and the therapeutic goal. 

## CONCLUSION

 Mini-PCNL appears to achieve stone-free and complication rates comparable to those of standard PCNL in pediatric patients while potentially offering advantages in terms of blood loss and length of hospital stay. However, these findings should be interpreted with caution because of the limited number of available studies, the inclusion of retrospective data, and heterogeneity in study quality. Further well-designed prospective randomized studies are needed to confirm these findings. 

## Data Availability

The data supporting the findings of this systematic review and meta-analysis, including the extracted dataset and statistical analysis files, are available upon reasonable request from the corresponding author, José Bessa Júnior.

## References

[1] European Association of Urology EAU Guidelines on Urolithiasis – INTRODUCTION – Uroweb [Internet].

[2] Nouralizadeh A, Sharifiaghdas F, Pakmanesh H (2018). Fluoroscopy-free ultrasonography-guided percutaneous nephrolithotomy in pediatric patients: a single-center experience. World J Urol.

[3] Özden E, Mercimek MN, Yakupoğlu YK, Özkaya O, Sarikaya Ş (2011). Modified Clavien classification in percutaneous nephrolithotomy: assessment of complications in children. J Urol.

[4] Özden E, Şahin A, Tan B, Doğan HS, Eren MT, Tekgül S (2008). Percutaneous renal surgery in children with complex stones. J Pediatr Urol.

[5] Karatag T, Tepeler A, Silay MS (2015). A comparison of 2 percutaneous nephrolithotomy techniques for the treatment of pediatric kidney stones of sizes 10-20 mm: microperc vs miniperc. Urology.

[6] Ruhayel Y, Tepeler A, Dabestani S (2017). Tract sizes in miniaturized percutaneous nephrolithotomy: a Systematic review from the European Association of Urology Urolithiasis Guidelines Panel. Eur Urol.

[7] Mykoniatis I, Pietropaolo A, Pyrgidis N (2022). Mini percutaneous nephrolithotomy versus standard percutaneous nephrolithotomy for the management of renal stones over 2 cm: a systematic review and meta-analysis of randomized controlled trials. Minerva Urol Nephrol.

[8] Sharma G, Sharma A, Devana SK, Singh SK (2022). Mini versus standard percutaneous nephrolithotomy for the management of renal stone disease: systematic review and meta-analysis of randomized controlled trials. Eur Urol Focus.

[9] Riva JJ, Malik KMP, Burnie SJ, Endicott AR, Busse JW (2012). What is your research question? An introduction to the PICOT format for clinicians. J Can Chiropr Assoc.

[10] Clavien PA, Barkun J, Oliveira ML (2009). The Clavien–Dindo classification of surgical complications: five-year experience. Ann Surg.

[11] Luo D, Wan X, Liu J, Tong T (2018). Optimally estimating the sample mean from the sample size, median, mid-range, and/or mid-quartile range. Stat Methods Med Res.

[12] Higgins JPT, Thompson SG, Deeks JJ, Altman DG (2003). Measuring inconsistency in meta-analyses. BMJ.

[13] Song G, Guo X, Niu G, Wang Y (2015). Advantages of tubeless mini-percutaneous nephrolithotomy in the treatment of preschool children under 3 years old. J Pediatr Surg.

[14] Kumar N, Yadav P, Kaushik VN (2023). Mini-versus standard percutaneous nephrolithotomy in pediatric population: a randomized controlled trial. J Pediatr Urol.

[15] Akdogan N, Deger M, Yilmaz IO (2024). Is percutaneous nephrolithotomy effective and safe in infants younger than 2 Years old? Comparison of mini standard percutaneous nephrolithotomy. J Pediatr Urol.

[16] Karkin K, Aydamirov M, Aksay B (2024). Comparison of two percutaneous nephrolithotomy methods for the treatment of pediatric kidney stones: Mini-PCNL and standard PCNL. Arch Ital Urol Androl.

[17] Mahmood SN, Aziz BO, Tawfeeq HM, Fakhralddin SS (2019). Mini-versus standard percutaneous nephrolithotomy for treatment of pediatric renal stones: is smaller enough?. J Pediatr Urol.

[18] Qin P, Zhang D, Huang T, Fang L, Cheng Y (2022). Comparison of mini percutaneous nephrolithotomy and standard percutaneous nephrolithotomy for renal stones > 2 cm: a systematic review and meta-analysis. Int Braz J Urol.

