# Supplementary material for: Standard versus miniaturized percutaneous nephrolithotomy in pediatric patients: a systematic review and meta-analysis
Source: Sao Paulo Med J. 2026 Sep 18;144(4):e20253486. doi: 10.1590/1516-3180.2025.3486.29062026 (PMC13588546; doi:10.1590/1516-3180.2025.3486.29062026)
Supplement: Supplementary file 1 [file 1806-9460-spmj-144-04-e20253486-md1.docx]

Standard versus miniaturized percutaneous nephrolithotomy in pediatric patients: a systematic review with meta-analysis

Fernanda Santos da Anunciação^I^, João Lucas de Magalhães Leal Moreira^II^, Caio Cezar Ferreira Fraga^III^, Caio Vinicius Suartz^IV^, Ricardo Brianezi Tiraboschi^V^, José Bessa Júnior^VI^, Roberto Iglesias Lopes^VII^

Universidade Estadual de Feira de Santana (UEFS), Feira de Santana (BA), Brazil

^I^Undergraduate Student, Escola de Medicina, Universidade Estadual de Feira de Santana (UEFS), Feira de Santana (BA), Brazil.

 <https://orcid.org/0009-0008-1685-7726>

^II^Undergraduate Student, Escola de Medicina, Universidade Estadual de Feira de Santana (UEFS), Feira de Santana (BA), Brazil.

 <https://orcid.org/0009-0008-4457-6091>

^III^Undergraduate Student, Escola de Medicina, Universidade Estadual de Feira de Santana (UEFS), Feira de Santana (BA), Brazil.

 <https://orcid.org/0009-0002-9323-4940>

^IV^PhD, MD. Assistant Professor, Urology Department, Northern Ontario School of Medicine, Thunder Bay (ON), Canada.

 <https://orcid.org/0000-0002-1364-5508>

^V^PhD, MD. Full Professor, Divisão de Urologia, Universidade Estadual de Feira de Santana (UEFS), Feira de Santana (BA), Brazil.

 <https://orcid.org/0000-0001-8502-1983>

^VI^PhD, MD. Full Professor, Divisão de Urologia, Universidade Estadual de Feira de Santana (UEFS), Feira de Santana (BA), Brazil.

 <https://orcid.org/0000-0003-4833-4889>

^VII^PhD, MD. Assistant Professor, Divisão de Urologia, Faculdade de Medicina, Universidade de São Paulo (FMUSP), São Paulo (SP), Brazil.

 <https://orcid.org/0000-0002-6636-7679>

**KEYWORDS (MeSH terms):**

Urology.

Pediatrics.

Urolithiasis.

Nephrolithotomy, Percutaneous.

**AUTHOR’S KEYWORDS:**

Pediatric Endourology.

Miniaturized Surgery.

Percutaneous Renal Access.

Perioperative Complications.

Clavien-Dindo Classification.

Renal Stone Surgery.

Supplementary material

**Suppl. Figure 1.** RoB2.

**Suppl. Figure 2.** Robins-I.

**Suppl. Figure 3.** Leave one out operation time, wasn’t found the source of heterogenety.

No significant difference between the groups.

**Suppl. Figure 4.** Complications: Clavien grade 2 – blood transfusion, nephrostomy site leak.

No significant difference between the groups.

**Suppl. Figure 5.** Complications: Clavien grade 3 – Nephropleural fistula.

No significant difference between the groups.

**Suppl. Figure 6.** Complications: Clavien grade 4 – Organ disfunction (single or multiple).

No significant difference between the groups.

**Suppl. Figure 7.** Blood transfusion.

No significant difference between the groups.

**Suppl. Figure 8.** Hospitalization stay.

Wasn’t found the source of heterogeneity.

**Suppl. Figure 9.** Leave one out of blood transfusion.

No significant difference between the groups.

**Suppl. Figure 10.** Use of ureteral stent (double j stent).

No significant difference between the groups.

**Suppl. Figure 11.** Hemoglobin drop.

**Suppl. Figure 12.** Leave-one-out hemoglobin drop.

No significant difference between the groups.

**Suppl. Figure 13.** Single puncture.

Significant difference between the groups favor SPCNL RR 2.946, 95% CI [1.175; 7.3.389], p < 0.05, I² = 0%.

**Suppl. Figure 14.** Multiple puncture.
